# Abdominal ultrasound activates afferent vagus nerve fibers and induces anti-inflammatory effects

**DOI:** 10.1073/pnas.2518969123

**Published:** 2026-02-11

**Authors:** Kotaro Shimoyama, Mamoru Tanida, Jun Aruga, Tomohiro Furusato, Chia-Hsien Wu, Yasuna Nakamura, Daisuke Takahashi, Go Kanzaki, Atsuhiro Maeda, Takao Shioya, Nobuo Tsuboi, Chikara Abe, Takashi Yokoo, Ryusuke Umene, Tsuyoshi Inoue

**Affiliations:** ^a^Department of Physiology of Visceral Function and Body Fluid, Graduate School of Biomedical Sciences, Nagasaki University, Nagasaki 852-8523, Japan; ^b^Division of Nephrology and Hypertension, Department of Internal Medicine, The Jikei University School of Medicine, Tokyo 105-8461, Japan; ^c^Department of Physiology II, Kanazawa Medical University, Ishikawa 920-0293, Japan; ^d^Department of Medical Pharmacology, Nagasaki University Institute of Biomedical Sciences, Nagasaki 852-8523, Japan; ^e^Graduate School of Integrated Science and Technology, Nagasaki University, Nagasaki 852-8521, Japan; ^f^Department of Physiology, Faculty of Medicine, Saga University, Saga 849-8501, Japan; ^g^Department of Physiology, University of Fukui School of Medical Sciences, Fukui 910-1193, Japan

**Keywords:** ultrasound, cholinergic anti-inflammatory pathway, vagus nerve, macrophage, inflammation

## Abstract

Abdominal ultrasound has emerged as a noninvasive modality with immunomodulatory potential. Although its anti-inflammatory effects have been demonstrated in various disease models, the underlying mechanisms remain unclear. Previous studies suggest that ultrasound promotes anti-inflammatory macrophage polarization via α7 nicotinic acetylcholine receptor (α7nAChR) signaling in the spleen. However, the upstream events initiating this response have not been elucidated. Here, we demonstrate that abdominal ultrasound activates afferent vagal fibers and suppress systemic inflammation. In a murine model of lipopolysaccharide (LPS)-induced endotoxemia, abdominal ultrasound significantly reduced plasma TNF-α levels. This anti-inflammatory effect was attenuated by subdiaphragmatic vagotomy (SDVx) or afferent vagal blockade. Electrophysiological recordings revealed increased cervical vagus nerve activity during ultrasound stimulation, which was eliminated by intraperitoneal lidocaine, confirming activation of abdominal sensory afferents. Furthermore, abdominal ultrasound induced c-Fos expression in the nucleus tractus solitarius (NTS), consistent with central activation via vagal afferent input. These findings provide direct mechanistic evidence that abdominal ultrasound stimulates afferent vagal pathways.

Ultrasound is widely used in clinical settings for diagnostic imaging; however, recent studies have highlighted its potential as a therapeutic tool. In preclinical models, pulsed abdominal ultrasound has been shown to confer anti-inflammatory and organ-protective effects. A landmark study in 2013 first demonstrated that abdominal ultrasound attenuates acute kidney injury (AKI) in a murine ischemia reperfusion model (1). Since then, similar therapeutic benefits have been reported across a broad range of pathological contexts (2–5). These effects are thought to arise from the polarization of immune cells—particularly macrophages—toward an anti-inflammatory phenotype. A pivotal molecule in this process is the α7 nicotinic acetylcholine receptor (α7nAChR), expressed on macrophages, which serves as a key switch for inducing the anti-inflammatory (M2-like) phenotype. However, the upstream signals by which ultrasound stimulation activates α7nAChR have remained unclear, posing a major barrier to optimizing this approach for clinical translation.

α7nAChR is a well-established downstream effector of the cholinergic anti-inflammatory pathway (CAP), a neuroimmune reflex circuit initiated by vagus nerve stimulation (VNS) (6). The canonical CAP involves the following steps: activation of the vagus nerve, engagement of the splenic sympathetic nerve, activation of splenic T cells that release acetylcholine (ACh), and subsequent activation of α7nAChR on splenic macrophages, which then shift toward an anti-inflammatory phenotype. Based on this framework, any of these nodes could serve as a target for ultrasound-induced neuromodulation. Given the dense innervation of the abdominal viscera by the vagus nerve, prior evidence that ultrasound can stimulate neurons through mechanical forces (7), and the critical role of afferent vagal input in initiating CAP (8), we hypothesized that abdominal ultrasound activates vagal afferent fibers to trigger CAP and suppress systemic inflammation. In this study, we sought to test this hypothesis using a combination of electrophysiological recordings, c-Fos immunohistochemistry, and genetic approaches.

## Results

**Fig. 1.**
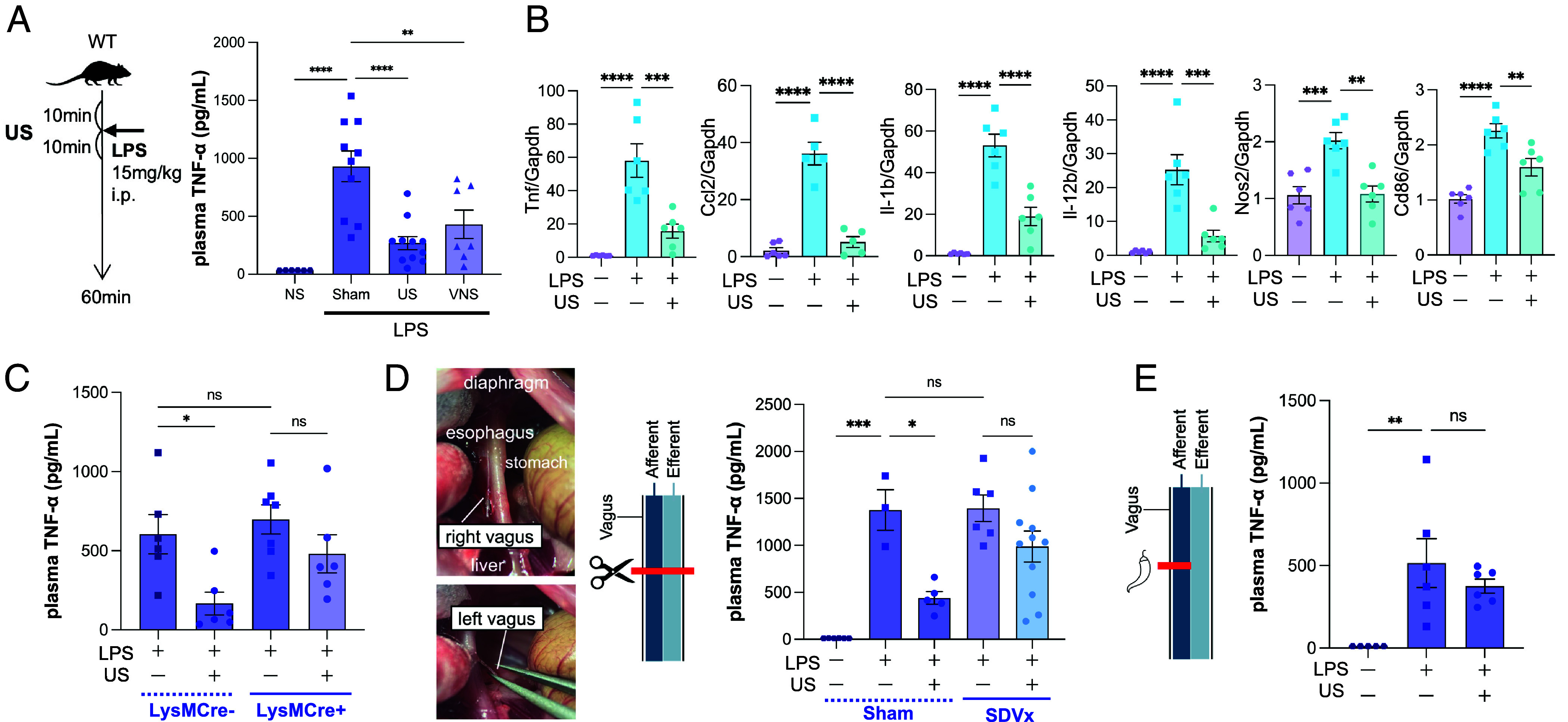
Abdominal ultrasound stimulation elicits anti-inflammatory effects via a CAP-like mechanism involving the vagus nerve. (*A*) Plasma TNF-α levels were elevated after intraperitoneal LPS injection but were reduced following abdominal ultrasound (US) stimulation. The magnitude of suppression was comparable to that observed with cervical vagus nerve stimulation. (*B*) Quantitative real-time PCR analysis of spleen revealed that proinflammatory cytokines (Tnf, Ccl2, Il-1b, Il-12b) and M1 macrophage markers (Nos2, Cd86) were suppressed by US treatment. (*C*) In macrophage-specific α7nAChR knockout mice (LysM-Cre:α7nAChR^flox^), TNF-α reduction was observed in Cre– mice following US stimulation, whereas this effect was attenuated in Cre+ mice. (*D*) Subdiaphragmatic vagotomy (SDVx) was performed by transecting the *Left* and *Right* vagus nerves along the esophagus. In the Sham operation group (laparotomy only), ultrasound exerted an anti-inflammatory effect, whereas in the SDVx group, the effect of ultrasound was attenuated. (*E*) In mice treated with capsaicin on both cervical vagus nerves to selectively block afferent vagal fibers, US failed to suppress inflammation.

We first evaluated the anti-inflammatory effects of ultrasound in a mouse model of endotoxemia. C57BL/6J mice were subjected to 10 min of abdominal ultrasound stimulation (Burst Mode, 14 MHz), followed by intraperitoneal injection of lipopolysaccharide (LPS; 15 mg/kg), and then an additional 10 min of ultrasound stimulation. Plasma TNF-α levels were significantly reduced following ultrasound treatment, comparable to the reduction observed in mice that received electrical cervical vagus nerve stimulation (VNS) (Fig. 1*A*). To assess changes in immune gene expression, we performed quantitative real-time PCR on spleen tissue. Ultrasound stimulation significantly suppressed the mRNA expression of proinflammatory cytokines and M1 macrophage markers (Fig. 1*B*), indicating activation of an anti-inflammatory response. Notably, in macrophage-specific α7nAChR knockout mice (LysM-Cre:α7nAChR^flox^) (9), the response to ultrasound was attenuated (Fig. 1*C*). This finding suggests the involvement of CAP mediated by α7nAChR on macrophages. To determine whether the vagus nerve mediates this effect, we employed two surgical models to disrupt vagal signaling. In mice that underwent subdiaphragmatic vagotomy (SDVx), the TNF-α–lowering effect of ultrasound was attenuated. (Fig. 1*D*). Similarly, in mice treated with capsaicin on the vagus nerve—a procedure that impairs afferent vagal fibers (10)—the effect of ultrasound was diminished (Fig. 1*E*). These findings suggest the involvement of vagal afferent pathways, and this was further examined by performing vagal nerve activity recordings.

**Fig. 2.**
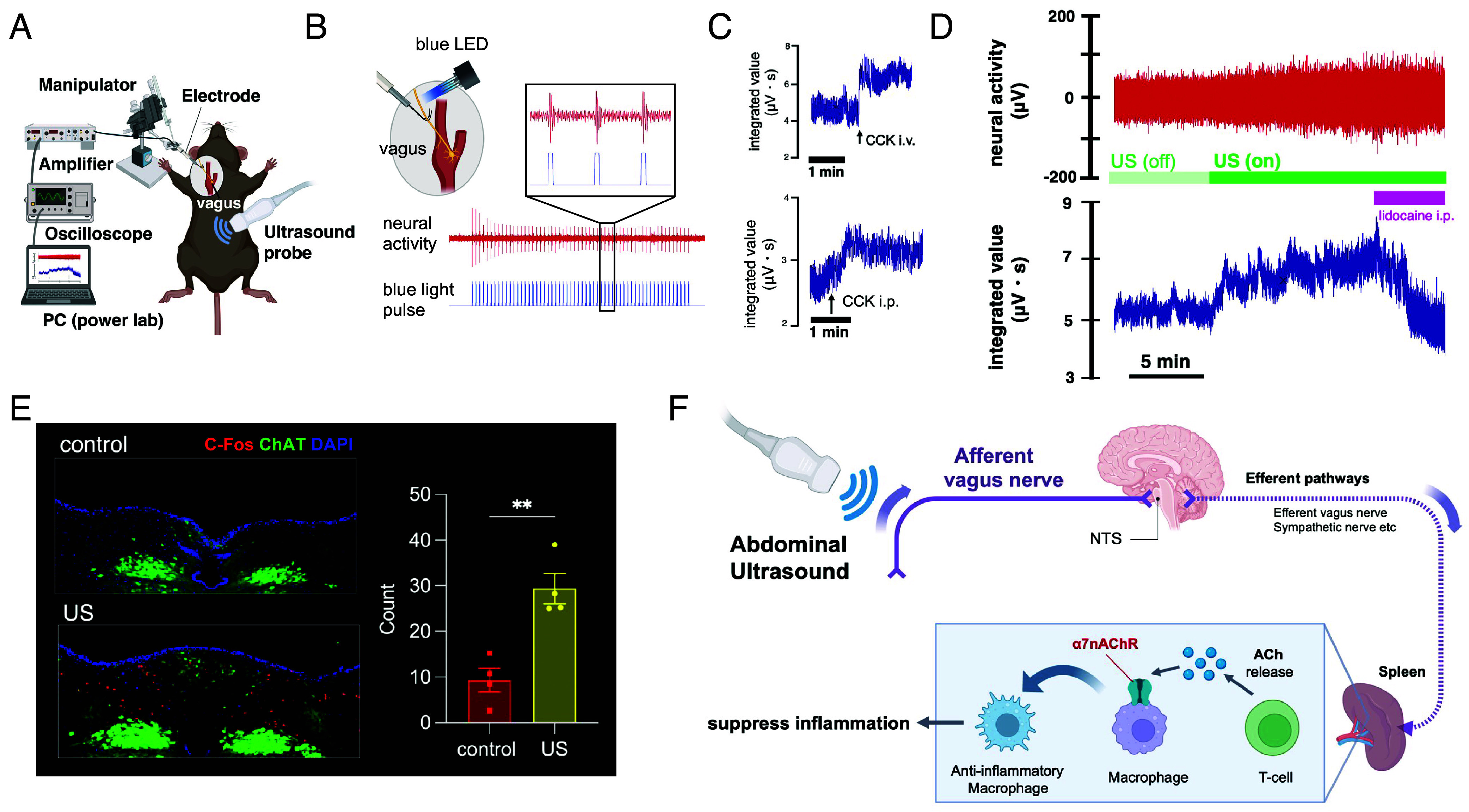
Abdominal ultrasound activates afferent vagal signaling and central autonomic pathways. (*A*) Schematic diagram of the cervical vagus nerve recording setup. (*B*) Validation of the recording system using optogenetics: blue light stimulation of the cervical vagus nerve in ChATCre-ChR2 mice evoked compound action potentials, confirming neural activation. (*C*) Intravenous or intraperitoneal administration of cholecystokinin (CCK), a known vagal activator, increased vagus nerve activity, further validating the recording setup. (*D*) Abdominal ultrasound (US) stimulation significantly increased cervical vagus nerve activity. This activation was abolished by intraperitoneal administration of lidocaine, indicating that the signal originates from abdominal afferents. (*E*) Immunohistochemical analysis revealed increased c-Fos expression in the nucleus tractus solitarius (NTS) 90 min after US stimulation, consistent with activation of vagal afferents projecting to the NTS. (*F*) Schematic illustration of the proposed mechanism by which abdominal ultrasound activates the cholinergic anti-inflammatory pathway (CAP).

To directly monitor vagus nerve activity during ultrasound stimulation, we established an electrophysiological recording system. Hook electrodes were placed on the left cervical vagus nerve, and neural signals were amplified and recorded in real time (Fig. 2*A*). To validate the system, we used ChATCre–ChR2 mice, in which channelrhodopsin-2 is selectively expressed in cholinergic neurons (11, 12). Optogenetic stimulation of the left vagus nerve elicited compound action potentials in response to blue light, serving as a methodological validation of our recording system rather than evidence for afferent mechanisms (Fig. 2*B*). Additionally, intravenous or intraperitoneal injection of cholecystokinin (CCK), a known activator of vagal afferents, induced neural activity, further validating the system (Fig. 2*C*). Ultrasound probe placement without activation did not elicit vagus nerve activity. In contrast, application of ultrasound resulted in a robust increase in vagus nerve activity, which was abolished by intraperitoneal administration of lidocaine (Fig. 2*D*), indicating that the response originates from abdominal afferents. To examine whether this vagal activation reaches the brainstem, we performed immunohistochemistry for c-Fos, a marker of neuronal activation, in the nucleus tractus solitarius (NTS)—the primary brainstem nucleus receiving vagal afferent input. Abdominal ultrasound significantly increased c-Fos expression in the NTS (Fig. 2*E*), demonstrating that vagal afferent activation by ultrasound is transmitted to central autonomic circuits.

## Discussion

Previous studies have demonstrated that stimulation of vagal afferent pathways can elicit anti-inflammatory effects (8, 11, 12). In the present study, we show that abdominal ultrasound activates vagal afferent signaling and induces anti-inflammatory responses (Fig. 2*F*). A limitation of our study is the use of capsaicin to inhibit vagal afferents. Capsaicin ablates TRPV1-expressing fibers and is often used as an afferent-targeting approach; however, a subset of vagal efferent neurons also expresses TRPV1, and these fibers may likewise be affected (13, 14). Therefore, our findings do not establish that vagal afferents represent the exclusive pathway mediating the anti-inflammatory effects of ultrasound. In addition, multiple downstream pathways beyond vagal afferents could contribute to these responses, and further studies will be required to delineate these mechanisms.

The organ-protective effects of ultrasound have attracted considerable attention, with reported benefits not only in acute diseases but also in chronic conditions such as arthritis (3), and ultrasound effects on the human spleen region have already been documented (15). Moving toward clinical application, it will be necessary to optimize parameters such as ultrasound intensity, frequency, location, and the attenuation effect through the skin.

## Materials and Methods

Experimental methods are provided in *SI Appendix*.

## Supplementary Material

Appendix 01 (PDF)

## Data Availability

All study data are included in the article and/or *SI Appendix*.
