# Supplementary material for: Abdominal ultrasound activates afferent vagus nerve fibers and induces anti-inflammatory effects
Source: Proc Natl Acad Sci U S A. 2026 Feb 11;123(7):e2518969123. doi: 10.1073/pnas.2518969123 (PMC12912971; doi:10.1073/pnas.2518969123)
Supplement: Supplementary file 1 — Appendix 01 (PDF) [file pnas.2518969123.sapp.pdf]

PNAS

Supporting Information for

Abdominal ultrasound activates afferent vagus nerve fibers and induces anti-inflammatory effects

Kotaro Shimoyama, Mamoru Tanida, Jun Aruga, Tomohiro Furusato, Chia-Hsien Wu, Yasuna Nakamura, Daisuke Takahashi, Go Kanzaki, Atsuhiro Maeda, Takao Shioya, Nobuo Tsuboi, Chikara Abe, Takashi Yokoo, Ryusuke Umene, Tsuyoshi Inoue

* Correspondence: Tsuyoshi Inoue
Email: ts-inoue@nagasaki-u.ac.jp

This PDF file includes:

Supporting Methods
SI References

Supporting Methods

Animals

Male mice (8–12 weeks of age, 20–25 g) were used in all experiments. Wild-type C57BL/6 mice were obtained from CLEA Japan, Inc. To generate macrophage-specific α7 nicotinic acetylcholine receptor (α7nAChR) knockout mice, Lysozyme M (LysM)-Cre mice were crossed with α7nAChR$^{flox/flox}$ mice (B6(Cg)-Chrna7$^{tm1.1Ehs}$/YakelJ, Jackson Laboratory, Bar Harbor, ME, USA: #026965) for several generations (1). LysM-Cre: α7nAChR^{flox} mice were genotyped using tail PCR according to the Jackson Laboratory protocol, with the MightyAmp Genotyping Kit (Takara Bio Inc., Shiga, Japan). Chat-ChR2 mice were created by crossing heterozygous Chat-ires-Cre mice (Jackson Laboratory: #028861) with homozygous Ai32 (RCL-ChR2(H134R)/EYFP) mice (Jackson Laboratory: #024109) (2). All surgical procedures and euthanasia were performed under general anesthesia using a mixture of 0.3 mg/kg medetomidine, 5 mg/kg butorphanol, and 4 mg/kg midazolam, administered intraperitoneally. Sepsis models were induced by intraperitoneal injection of lipopolysaccharide (LPS) from *Escherichia coli* O111:B4 (Cat# L2630, Sigma-Aldrich, Saint Louis, MO, USA) at a dose of 15 mg/kg body weight.

Sex as a Biological Variable

In our previous study using both wild-type and knockout mice (including LysM-Cre:α7nAChR^{flox} mice) in an LPS-induced sepsis model, we found no sex-related differences in inflammatory parameters, including plasma TNF-α levels (1). Therefore, only male mice were used in the present study.

Abdominal Ultrasound Protocol

Mice were anesthetized via intraperitoneal injection of a mixed anesthetic solution, and the abdominal area was thoroughly shaved and depilated using a commercial depilatory cream. The animals were placed in a prone position on a heating pad, and a rectal temperature probe was inserted to maintain the body temperature of 35.5 °C. The ultrasound probe (Nihon 3B Scientific, Niigata, Japan) was positioned in contact with the abdomen using an adequate amount of ultrasound gel (Aquasonic 100, Parker Laboratories, Fairfield, NJ). Ultrasound parameters were set based on previous studies demonstrating anti-inflammatory effects of abdominal ultrasound stimulation (3-5): mode, burst mode (square waveform); frequency, 14 MHz; amplitude, 1000 mA; burst length, 10 ms; voltage, 50 V; and pulse repetition frequency (PRF), 1 or 20 Hz. Ultrasound was applied for a total of 20 minutes, comprising 10 minutes before and 10 minutes after lipopolysaccharide (LPS) injection. Blood and spleen samples were collected 60 minutes after LPS administration. This protocol was adapted from a seminal study demonstrating cholinergic anti-inflammatory effects mediated by vagus nerve stimulation (VNS) (6).

Vagotomy and Selective Afferent Blockade

Bilateral subdiaphragmatic vagotomy was performed to generate vagotomized mice (7). Under anesthesia, a midline laparotomy was made to expose the stomach. The stomach was gently retracted posteriorly using an organ clip to expose the esophagus, and both vagus nerve trunks adjacent to the esophagus were transected using fine forceps. The abdominal wall was then sutured. Under this condition, since an abdominal incision was required to access the ultrasound target site, we also examined the effect of ultrasound in the Sham group, in which only laparotomy was performed. For selective afferent vagal blockade, 1% capsaicin was used. Under anesthesia, both cervical vagus nerves were exposed, and a strip of Parafilm was inserted underneath each nerve. Cotton balls soaked in 1% capsaicin solution were applied to the nerves and replaced every 10 minutes for a total exposure duration of 30 minutes (8). After treatment, the nerves were rinsed with 0.9% saline, and the incisions were closed. The 1% capsaicin solution was prepared as follows: 23.89 mg of capsaicin (030-11353, Fujifilm Wako, Japan) was dissolved in 240 µL of Tween 80 and sonicated for 5 minutes using a vortex and ultrasonic bath. Subsequently, 2150 µL of corn oil was added, and the mixture was sonicated again for 5 minutes.

Measurement of TNF-α

Plasma TNF-α levels were measured by ELISA using the TNF alpha Mouse Uncoated ELISA Kit with Plates (88-7324-22, Thermo Fisher Scientific, Waltham, MA), according to the manufacturer's instructions. Absorbance was read using a Synergy LX plate reader (BioTek Instruments, Winooski, VT).

Measurement of Spleen Cytokines by Real-Time PCR

Total RNA was extracted from one-quarter of the spleen using RNAiso Plus (9108, Takara Bio Inc., Shiga, Japan). Reverse transcription was performed using the PrimeScript RT Master Mix (RR036A, Takara Bio Inc., Shiga, Japan). The resulting cDNA was used as a template for quantitative real-time PCR, conducted with SYBR Green qPCR Master Mix (Thermo Fisher Scientific) on a CFX Duet Real-Time PCR System (Bio-Rad, Hercules, CA). Glyceraldehyde 3-phosphate dehydrogenase (Gapdh) was used as the internal control. Relative gene expression levels were calculated using the comparative cycle threshold (ΔΔCt) method. Primer sequences were as follows. *Gene name* (Forward 5'→3', Reverse 5'→3'): *Gapdh* (AGGTCGGTGTGAACGGATTTG, TGTAGACCATGTAGTTGAGGTCA); *Tnf* (GCCTCTTCTCATTCCTGCTTG, CTGATGAGAGGGAGGCCATT); *Ccl2* (GACCTTAGGGCAGATGCAGT, AGCTGTAGTTTTTGTCACCAAGC); *Il1b* (CCTTCCAGGATGAGGACATGA, AACGTCACACACCAGCAGGTT); *Il12b* (ATTACTCCGGACGGTTCACG, ACGCCATTCCACATGTCACT); *Nos2* (CACCTTGGAGTTCACCCAGT, ACCACTCGTACTTGGGATGC); *Cd86* (TCCAAGTTTTTGGGCAATGTC, CCTATGAGTGTGCACTGAGTTA)

Vagus Nerve Electrophysiological Recording

Vagus nerve action potentials were recorded based on a previously described protocol (8), with modifications to accommodate abdominal ultrasound (US) stimulation. Mice were anesthetized via intraperitoneal injection of a mixed anesthetic solution. Cervical and abdominal hair was shaved, and depilatory cream was applied to the abdominal area. The mice were placed prone on a surgical platform. A midline cervical incision was made, and the submandibular glands were retracted to expose the carotid artery and adjacent vagus nerve. A tracheotomy was performed, and a polyethylene tube was inserted to allow spontaneous breathing under oxygen-rich air. An intravenous line was established via the jugular vein for drug administration. To record afferent vagal signals, the vagus nerve was transected proximally (central side), and a bipolar stainless-steel electrode mounted on a micromanipulator was placed on the distal (peripheral) side. The nerve and electrode were embedded in silicone gel. To minimize respiratory and cardiac noise, the cervical vagus nerve—which contains both cardiac and pulmonary branches—was partially dissected, and a branch with minimal respiratory noise and no cardiac artifacts was selected. Neural signals were amplified (2000-fold amplification) and filtered (100–1000 Hz) using a bioamplifier (RR-1, CygnusTech, USA), displayed on an oscilloscope, and recorded in real time as analog-to-digital converted signals using a PowerLab system (4/26, ADInstruments, NSW, Australia). Vagus nerve activity was quantified as the rectified and integrated value derived from the raw voltage (μV). Nerve action potential recordings were conducted in three individual mice under identical experimental conditions to confirm reproducibility.

To validate the recording setup, we employed an optogenetic approach to selectively stimulate nerves using light (2, 7). Specifically, we used ChATCre-ChR2 mice, in which channelrhodopsin-2 (ChR2) is selectively expressed in cholinergic neurons. While recording from the peripheral side of the vagus nerve with a bipolar electrode, blue LED pulses (Doric Lenses, LEDFLS_465) were delivered to the central side (1 ms duration). Light stimulation evoked compound action potentials, with frequency-dependent responses observed at 1, 5, and 20 Hz (Main Figure 2B). To further validate the system, cholecystokinin (CCK; Peptide Institute, Inc.) at a dose of 8 μg/kg body weight was administered intravenously, resulting in increased vagus nerve activity. To determine whether the recorded cervical vagal activity includes afferent input from the abdominal cavity, CCK was also administered intraperitoneally. Although the response was delayed compared to intravenous administration, increased nerve activity was still observed (Main Figure 2C). Cervical vagus nerve activity was also recorded during abdominal ultrasound stimulation. The stimulation parameters were as follows: frequency, 14 MHz; current, 1000 mA; burst length,

10 ms; and pulse repetition frequencies (PRFs) of 1 Hz or 20 Hz. The PRF settings were selected based on previous electrical stimulation experiments conducted in our laboratory, and measurements were performed at both the lower and upper frequency bounds (1 and 20 Hz) (6, 9). To control for the mechanical pressure exerted by the probe itself, vagus nerve activity was compared between two conditions: probe placement without ultrasound emission and active ultrasound stimulation with the probe in place. Ultrasound stimulation increased vagus nerve activity (Main Figure 2E). To confirm that the recorded cervical nerve activity was mediated by afferent signals from the abdominal cavity, 0.2% lidocaine was administered intraperitoneally during ultrasound stimulation.

C-Fos Immunostaining

Ultrasound stimulation and subsequent tissue fixation, c-Fos immunostaining, and analysis were performed by different investigators. To ensure the accuracy of c-Fos quantification, the experiments were conducted in a single-blind manner. Ultrasound stimulation was applied to mice for 20 minutes. In the sham control group, the ultrasound probe was placed on the abdomen without emitting ultrasound. C-Fos staining of the medulla oblongata was performed as follows. Brain tissues were collected 90 minutes after stimulation. A total of 48 serial coronal cryosections (24 sections × 2 series), each 35 μm thick, were prepared per mouse. Sections were collected in PBS (without calcium and magnesium; PBS [–]) and stored in 12-well culture plates. From each series, the four sections closest to the Bregma –7.30 mm level (Paxinos and Franklin, 2001) were selected for immunostaining. Sections were blocked in PBS (–) containing 5% normal donkey serum and 0.3% Triton X-100 for 1 hour at room temperature (RT) with gentle agitation. Primary antibodies were then applied and incubated for 3 nights at 4 °C: recombinant rabbit anti-c-Fos (1:1000, Synaptic Systems, Cat# 226008) and goat polyclonal anti-ChAT (1:500, Millipore, Cat# AB144P). Following primary antibody incubation, sections were washed five times for 1 hour each in PBS (–) at RT with gentle agitation. Secondary antibodies (Alexa Fluor 594-conjugated donkey anti-rabbit IgG, 1:2000; Alexa Fluor 488-conjugated donkey anti-goat IgG, 1:2000) were applied overnight at 4 °C. After incubation, sections were washed five times in PBS (–), each for 1 hour to overnight. Finally, sections were mounted onto glass slides and coverslipped using VECTASHIELD mounting medium with DAPI (Vector Laboratories, Cat# H-1200). Images were acquired using the BZ-X710 microscope (Keyence) and were analyzed by particle analysis function of ImageJ software (https://imagej.net/ij/).

Statistical Analyses

Data are presented as mean ± standard error of the mean (SEM). Comparisons between two groups were performed using the Student's t-test. For comparisons among three or more groups, one-way or two-way analysis of variance (ANOVA) followed by Tukey's post hoc test was used. A P value of <0.05 was considered statistically significant. All statistical analyses were conducted using GraphPad Prism 10 (GraphPad Software, San Diego, CA, USA).

Study Approval

All animal experiments were conducted in accordance with the Guidelines for the Care and Use of Laboratory Animals and were approved by Nagasaki University.

SI References

1. Y. Nakamura *et al.*, Alpha 7 nicotinic acetylcholine receptors signaling boosts cell-cell interactions in macrophages effecting anti-inflammatory and organ protection. *Commun Biol* **6**, 666 (2023).
2. S. Tanaka *et al.*, Vagus nerve stimulation activates two distinct neuroimmune circuits converging in the spleen to protect mice from kidney injury. *Proceedings of the National Academy of Sciences of the United States of America* **118** (2021).
3. J. C. Gigliotti *et al.*, Ultrasound Modulates the Splenic Neuroimmune Axis in Attenuating AKI. *Journal of the American Society of Nephrology : JASN* **26**, 2470-2481 (2015).
4. J. C. Gigliotti *et al.*, Ultrasound prevents renal ischemia-reperfusion injury by stimulating the splenic cholinergic anti-inflammatory pathway. *Journal of the American Society of Nephrology : JASN* **24**, 1451-1460 (2013).
5. T. Inoue *et al.*, Non-canonical cholinergic anti-inflammatory pathway-mediated activation of peritoneal macrophages induces Hes1 and blocks ischemia/reperfusion injury in the kidney. *Kidney Int* **95**, 563-576 (2019).
6. L. V. Borovikova *et al.*, Vagus nerve stimulation attenuates the systemic inflammatory response to endotoxin. *Nature* **405**, 458-462 (2000).
7. C. Abe *et al.*, C1 neurons mediate a stress-induced anti-inflammatory reflex in mice. *Nature neuroscience* **20**, 700-707 (2017).
8. T. Teratani *et al.*, The liver-brain-gut neural arc maintains the T(reg) cell niche in the gut. *Nature* **585**, 591-596 (2020).
9. T. Inoue *et al.*, Vagus nerve stimulation mediates protection from kidney ischemia-reperfusion injury through alpha7nAChR+ splenocytes. *J Clin Invest* **126**, 1939-1952 (2016).
